# Supplementary material for: Defining strawberry shape uniformity using 3D imaging and genetic mapping
Source: Hortic Res. 2020 Aug 1;7:115. doi: 10.1038/s41438-020-0337-x (PMC7395166; doi:10.1038/s41438-020-0337-x)
Supplement: Supplementary file 1 — Defining Strawberry Shape Uniformity using 3D Imaging and Genetic Mapping [file 41438_2020_337_MOESM1_ESM.docx]

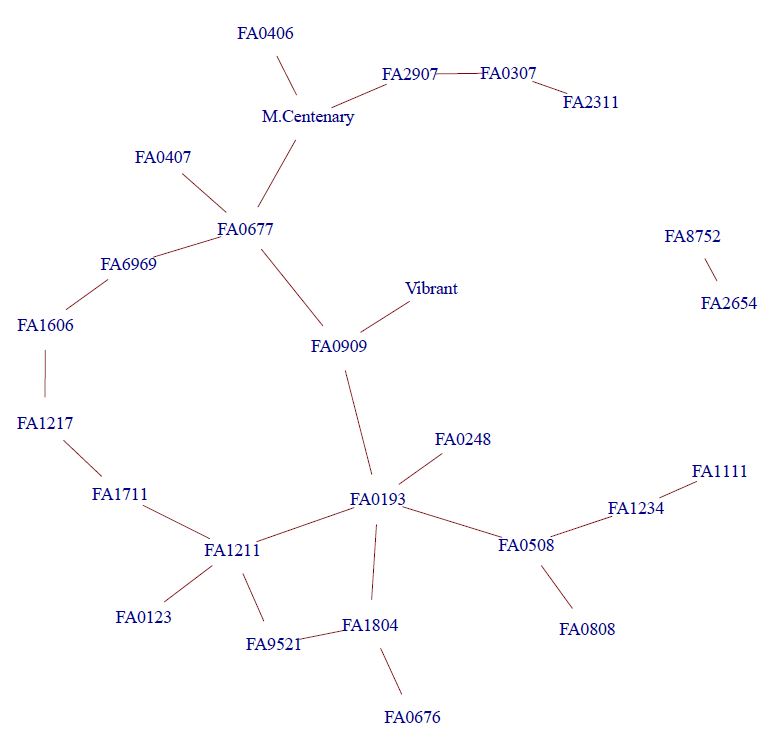


**Supplementary Figure 1.** A network of crosses conducted to generate the multiparental mapping population used in this study. Cultivars are represented by text, families are represented by lines.


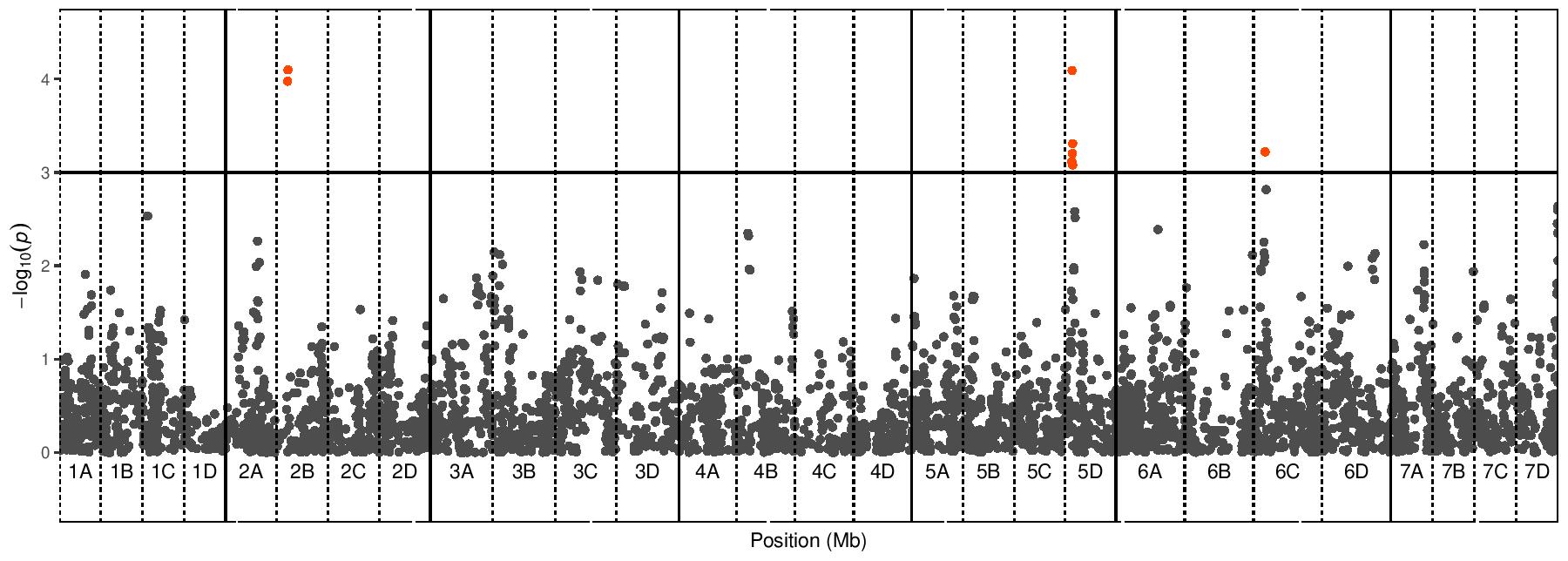


(a)


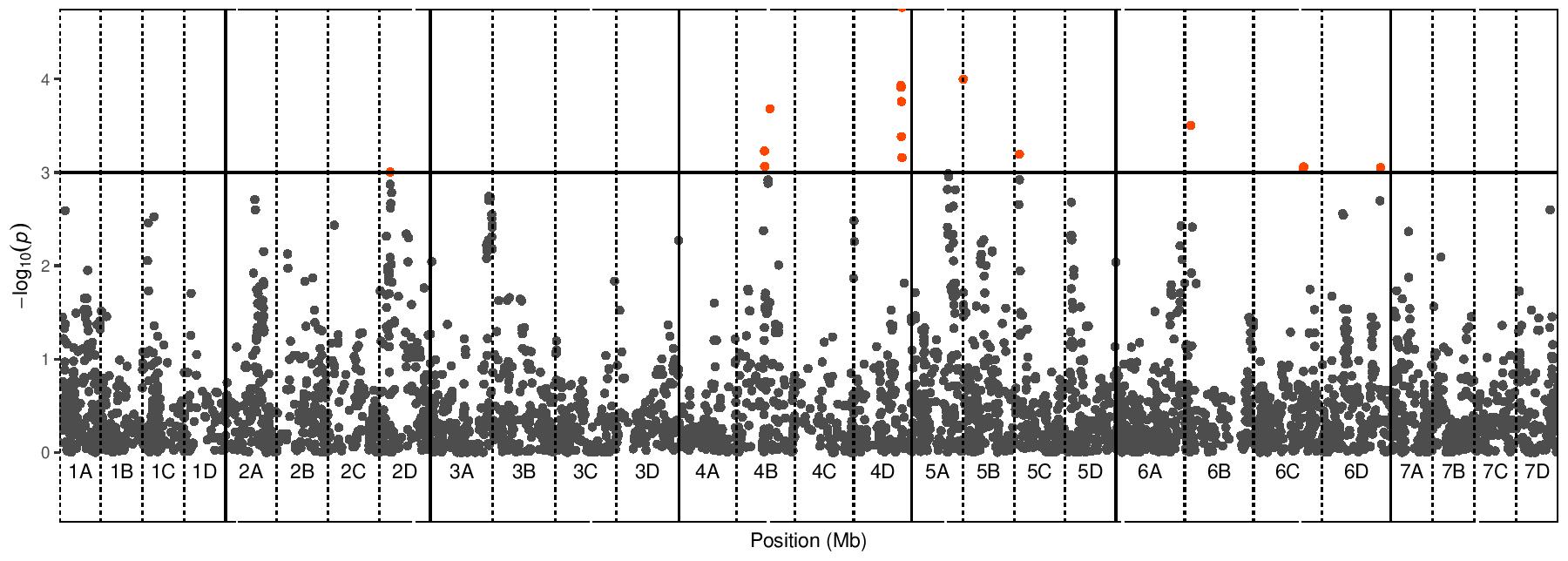


(b)

**Supplementary Figure 2.** Manhattan plots of (a) CV_A and (b) Max_C/Min_C.
